# Supplementary material for: The structural basis of translational control by eIF2 phosphorylation
Source: Nat Commun. 2019 May 13;10:2136. doi: 10.1038/s41467-019-10167-3 (PMC6513899; doi:10.1038/s41467-019-10167-3)
Supplement: Supplementary file 1 — Supplementary Information [file 41467_2019_10167_MOESM1_ESM.pdf]

Supplementary Material for:

The Structural Basis of Translational Control by eIF2 Phosphorylation

Adomavicius et al.

This file contains:

Supplementary Figures 1-8

Supplementary Tables 1-5

Supplementary Discussion

Supplementary References

Note: Supplementary Movies 1 and 2 are provided as separate files

Supplementary Figures

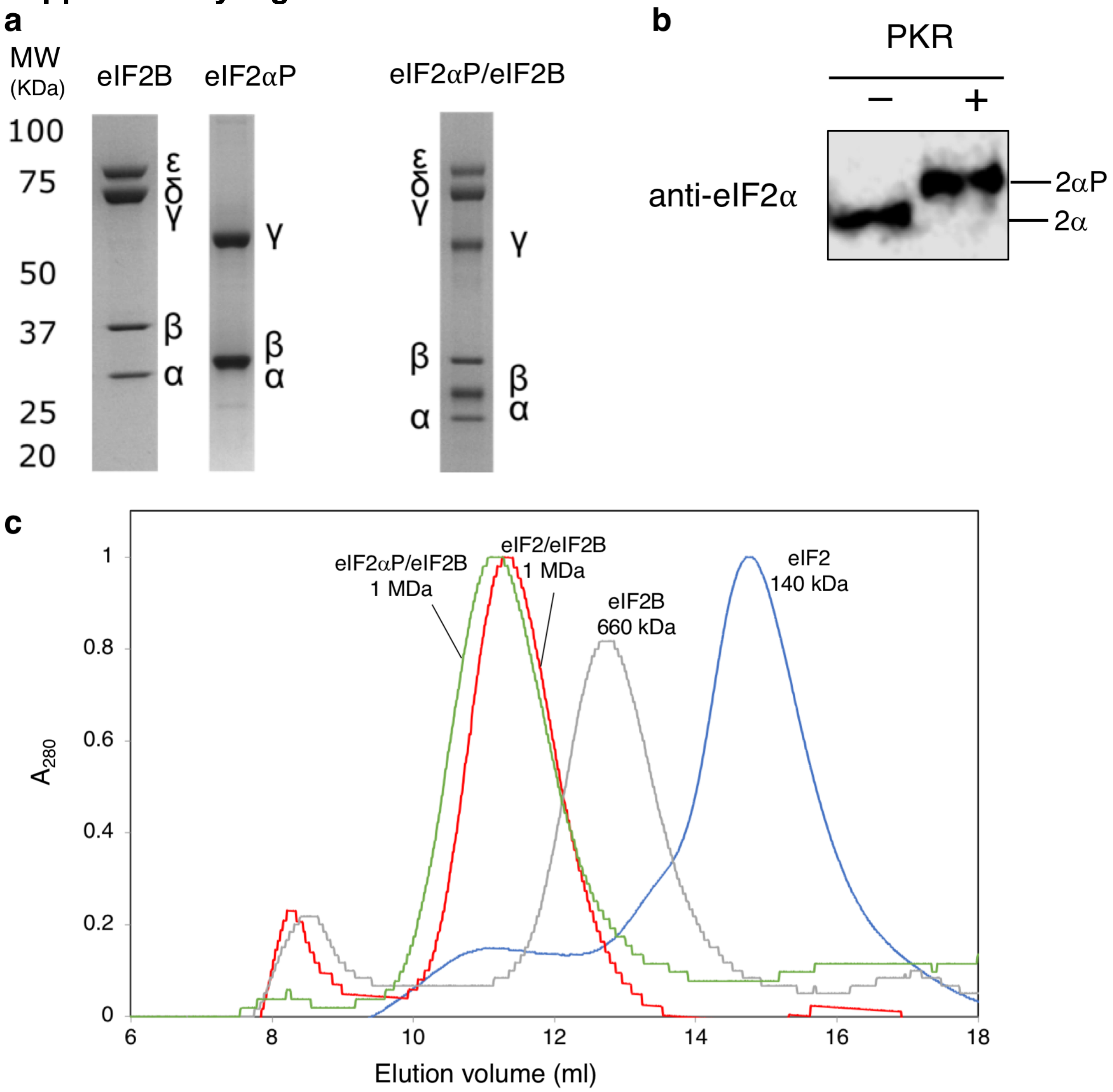

Supplementary Figure 1. Purified eIF2B, eIF2 and eIF2αP/eIF2B complexes

a, Coomassie blue stained SDS-PAGE of purified eIF2B, eIF2 as well as eIF2αP/eIF2B complexes following SEC.

b, Phos-Tag western blot of eIF2 treated with PKR to quantitatively phosphorylate it prior to complex formation with eIF2B.

c, Example SEC-MALLS traces of indicated complexes with peak MWs.

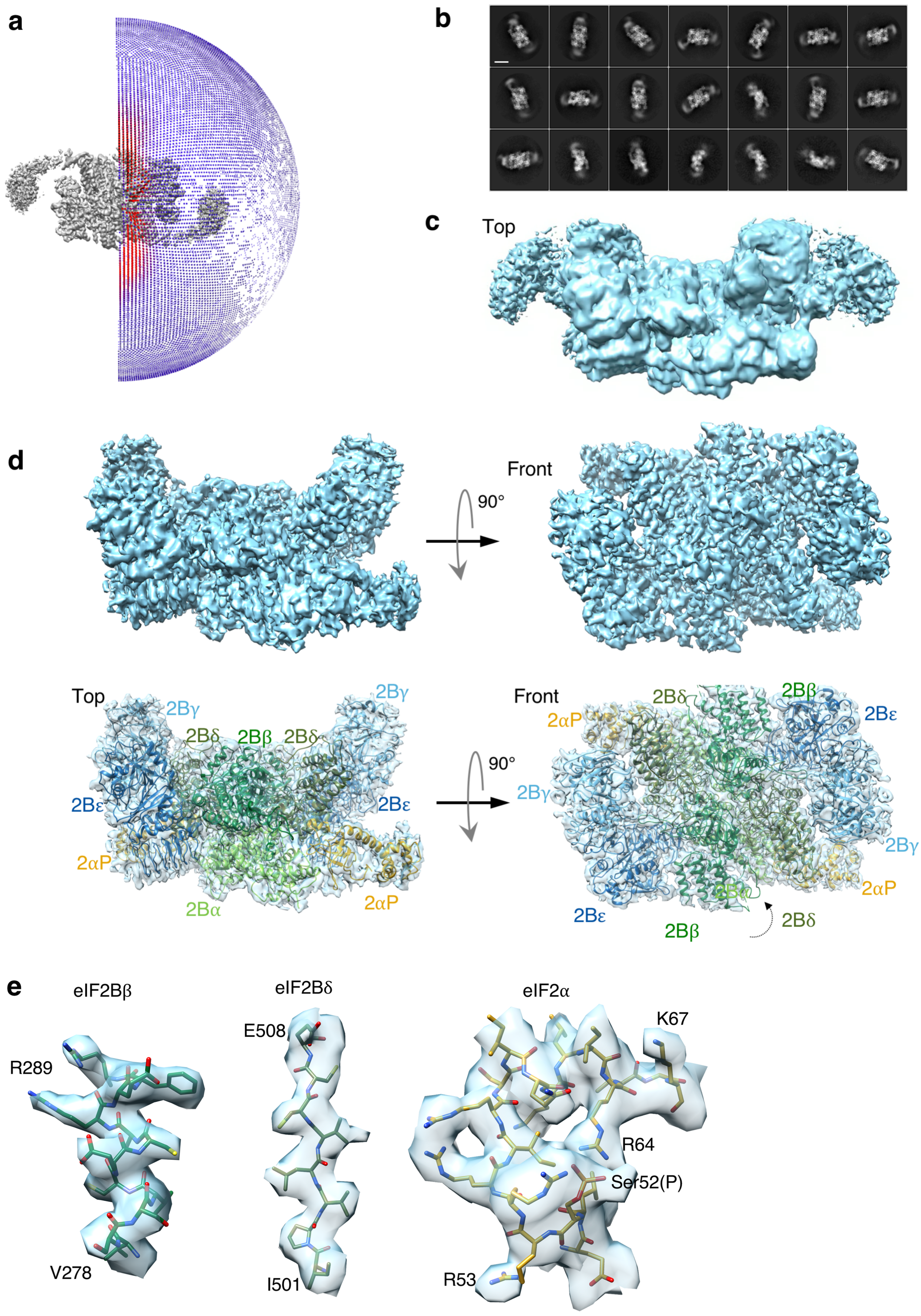

a
b
c
Top
d
Front
90°
Top
2Bγ
2Bδ
2Bβ
2Bδ
2Bγ
2Bε
2Bε
2αP
2Bα
2αP
Front
2Bδ
2Bβ
2αP
2Bε
2Bγ
2Bγ
2αP
2Bε
2Bβ
2Bδ
e
eIF2Bβ
R289
V278
eIF2Bδ
E508
I501
eIF2α
K67
R64
Ser52(P)
R53

f

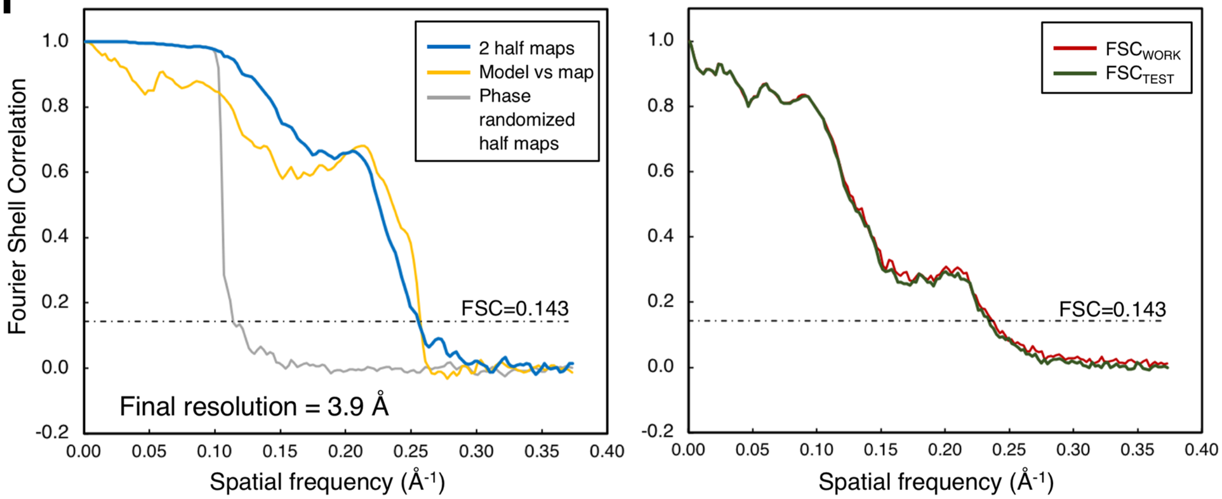

g

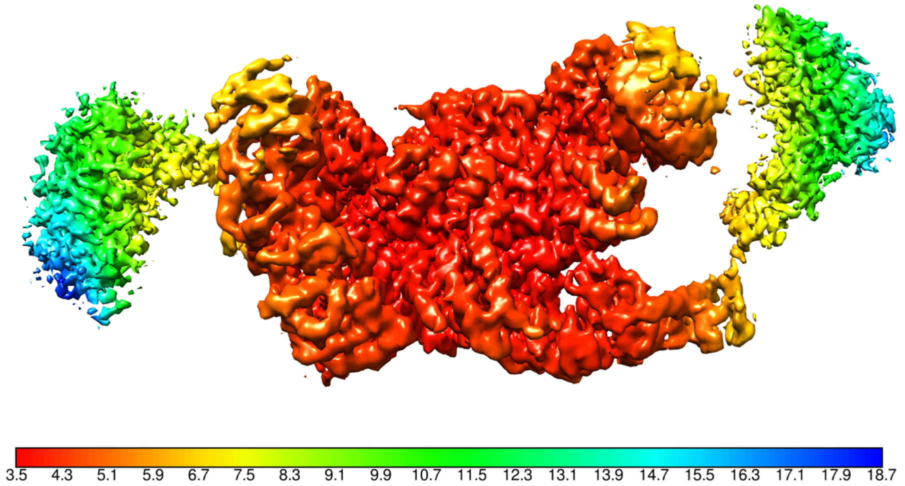

Supplementary Figure 2. A Cryo EM structure of the eIF2B-eIF2(αP) complex

a, Particle angle plot (Relion).

b, Representative 2D classes of eIF2αP/eIF2B. Scale bar is 100 Å.

c, Initial 3D map of the eIF2αP/eIF2B complex, top view. The length of the complex is ~280 Å. Though irregular in shape, it would be enclosed by a rectangular box of dimensions 280 x120 x 120 Å.

d, refined 3.9 Å map of the core complex (top) and modelled densities comprising eIF2Bα–ε decamer and two eIF2α$^{1\text{-}174}$ monomers (bottom). Top and front views shown.

e, Representative regions of eIF2αP/eIF2B density depicted as transparent isosurface with atomic model fitted.

f, Fourier Shell correlation (FSC) plots. Left. FSC computed using the gold-standard method (reported resolution by FSC criterion 0.143), FSC of final atomic model vs EM map, and FSC of half maps from refined phase randomized images. Right. Cross-validation FSCs show the weight applied in the atomic model refinement avoids over fitting.

g, Local resolution of the map shown as a colored surface applied to the map with local density re-scaling (using Locscale). The scalebar shows the local resolution (Å).

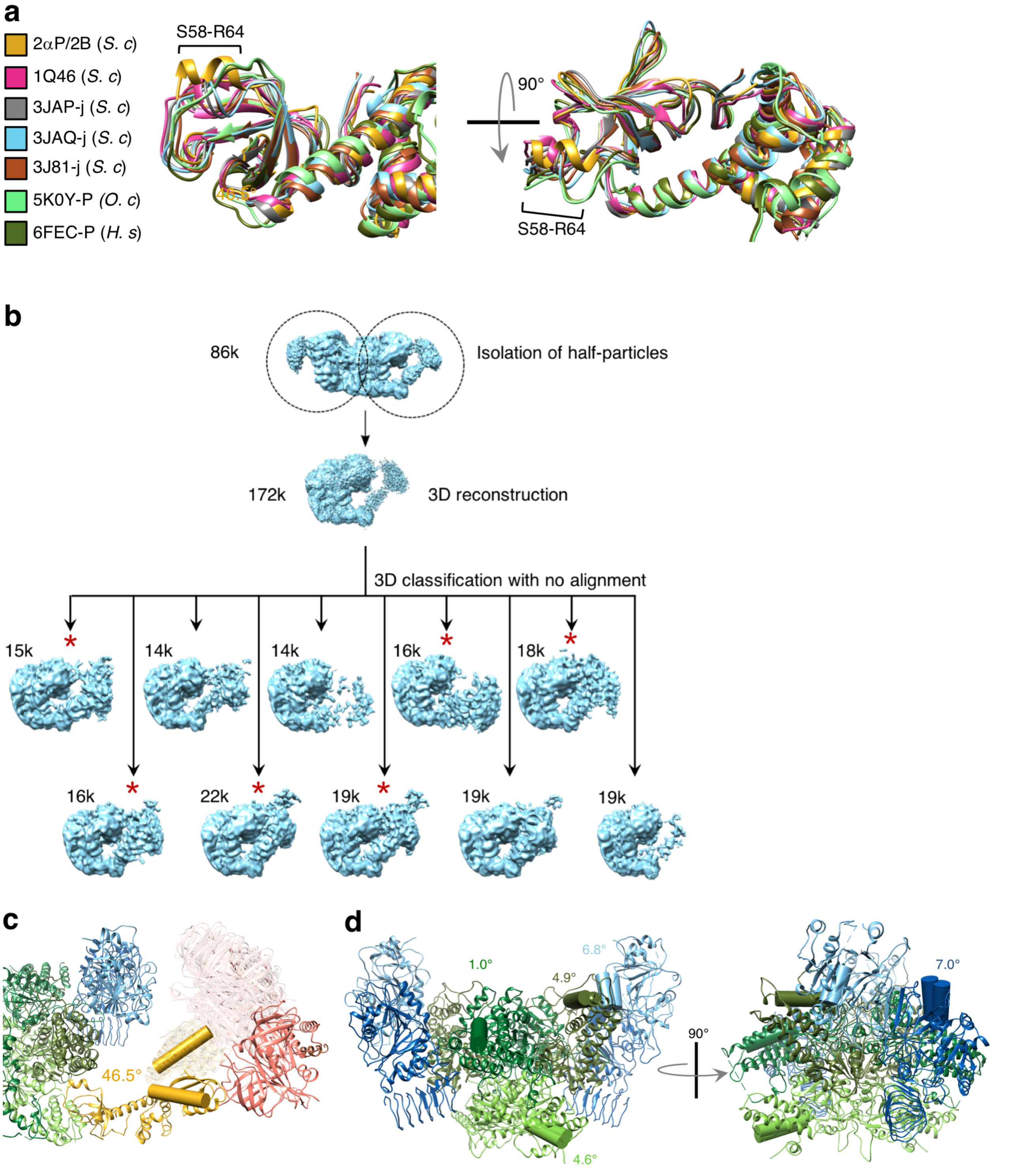

Supplementary Figure 3. Angle differences in eIF2a and eIF2B.

a, Alignment of eIF2α from eIF2αP/eIF2B with previous eIF2α structures from yeast (*S. c*), rabbit (*O. c*) and human (*H. s*) with PDB accession codes. Secondary structures shown only.
b, Isolation and reclassification of eIF2αP/eIF2B half particles into 10 classes(14-22,000 per class). Red asterisks denote subclasses used in Fig. 2 b-g.
c, Angle changes (°) observed in eIF2α domain 3 and noted in Fig. 2.
d, Angle changes (°) observed in eIF2B when *S. pombe* eIF2B crystal structure is compared with eIF2B/eIF2αP.

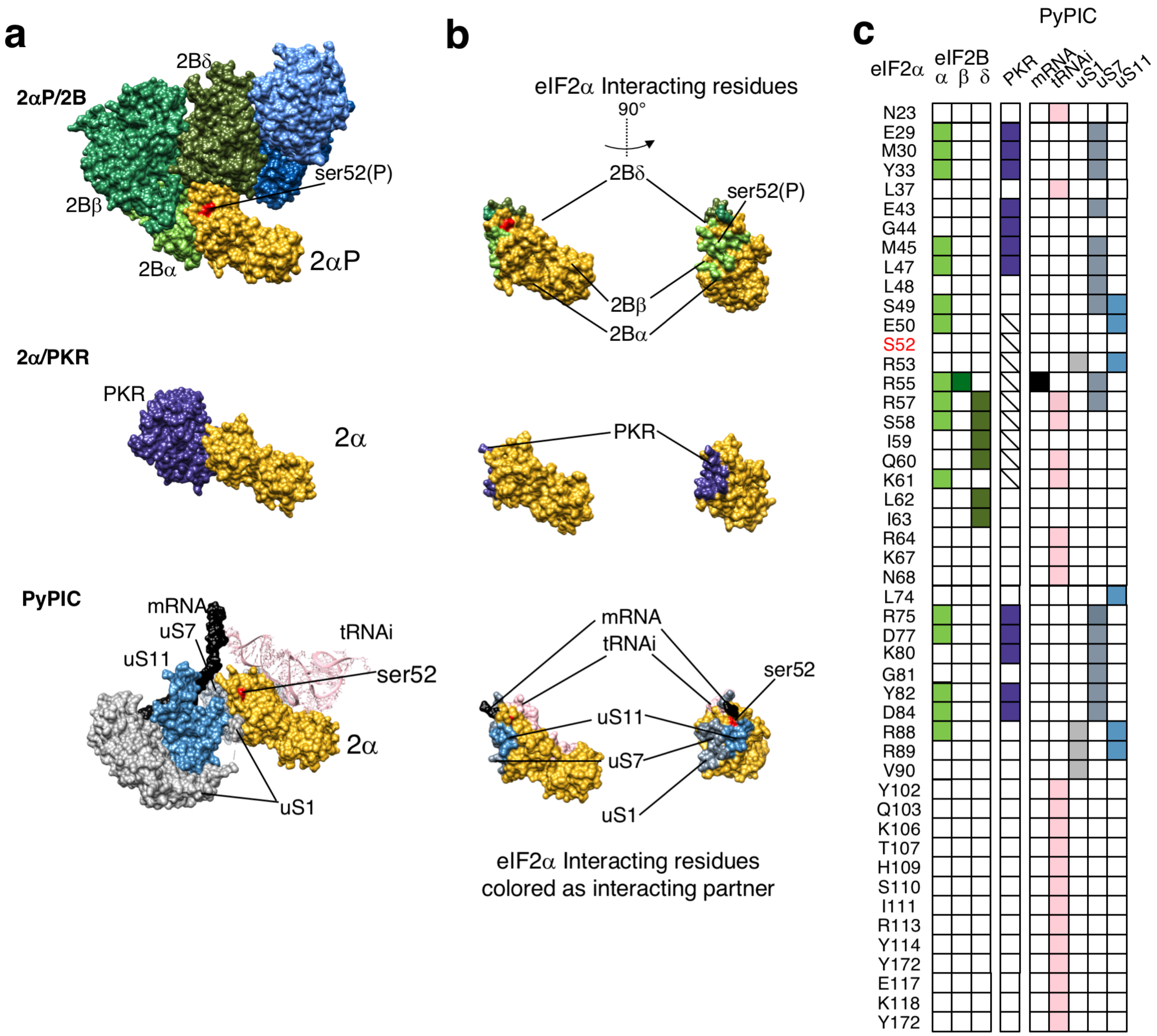

Supplementary Figure 4. Comparison of eIF2α interactions among different structures.

a, eIF2αP/eIF2B core interaction interface (top), eIF2α/PKR (PDB:2A1A, middle) and eIF2α/PyPIC interactions (PDB:3JAP, bottom).
b, eIF2α colored to show interacting surface residues in two orientations.
c, summary of eIF2α residue interactions across the three structures.

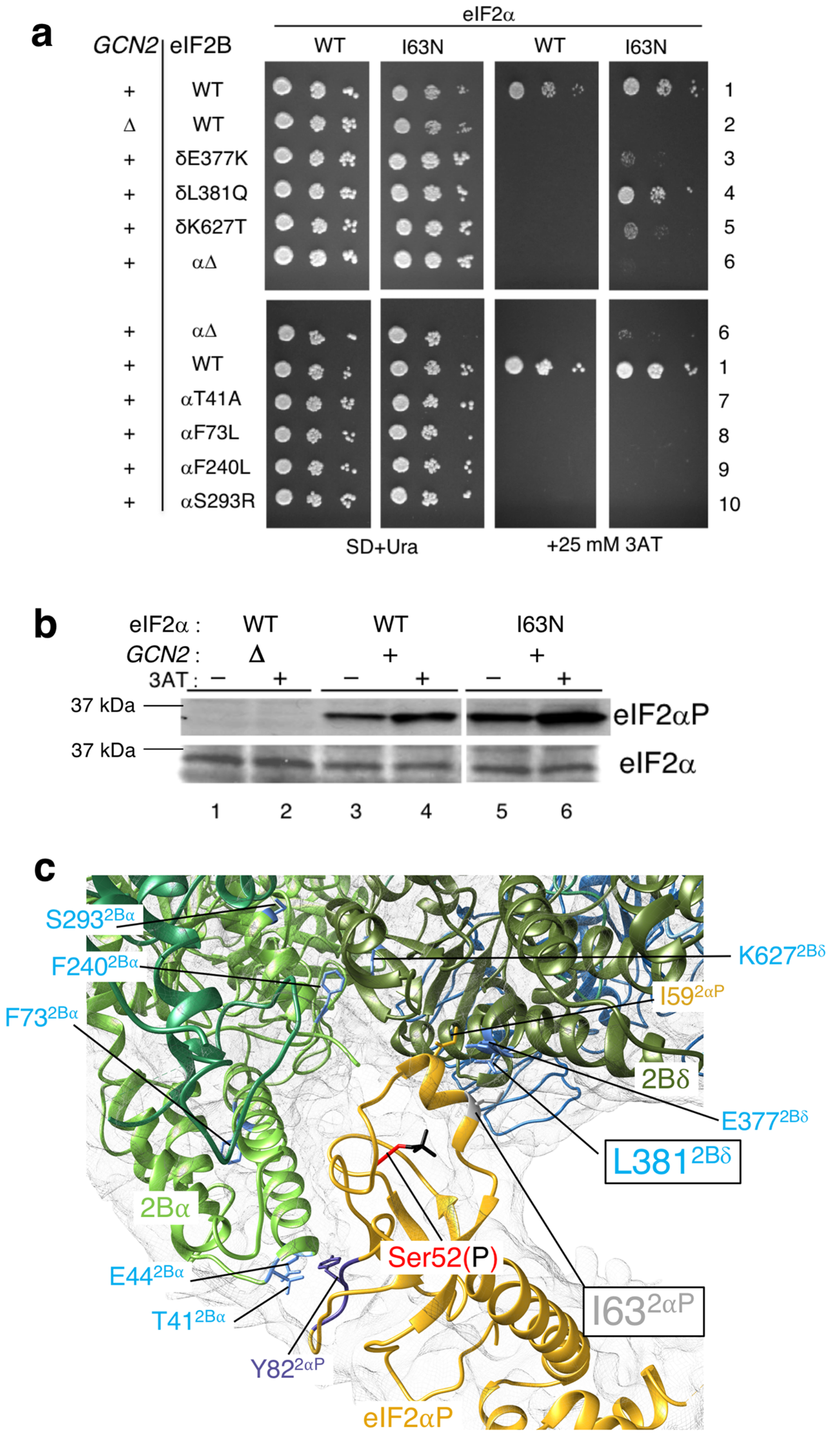

Supplementary Figure 5. eIF2α-I63N reverses the Gcn⁻ phenotype of eIF2Bδ-L381Q

a, Genetic analysis. I63N in eIF2α reverses Gcn⁻ of L381Q in eIF2Bδ but not other Gcn⁻ alleles tested.
b, eIF2α I63N is a substrate for Gcn2.
c, Structure shows that eIF2α-I63 and eIF2Bα-L381 are within 4.2 Å. Other labelled residues are those mutated in panel **a** or referred to in the text.

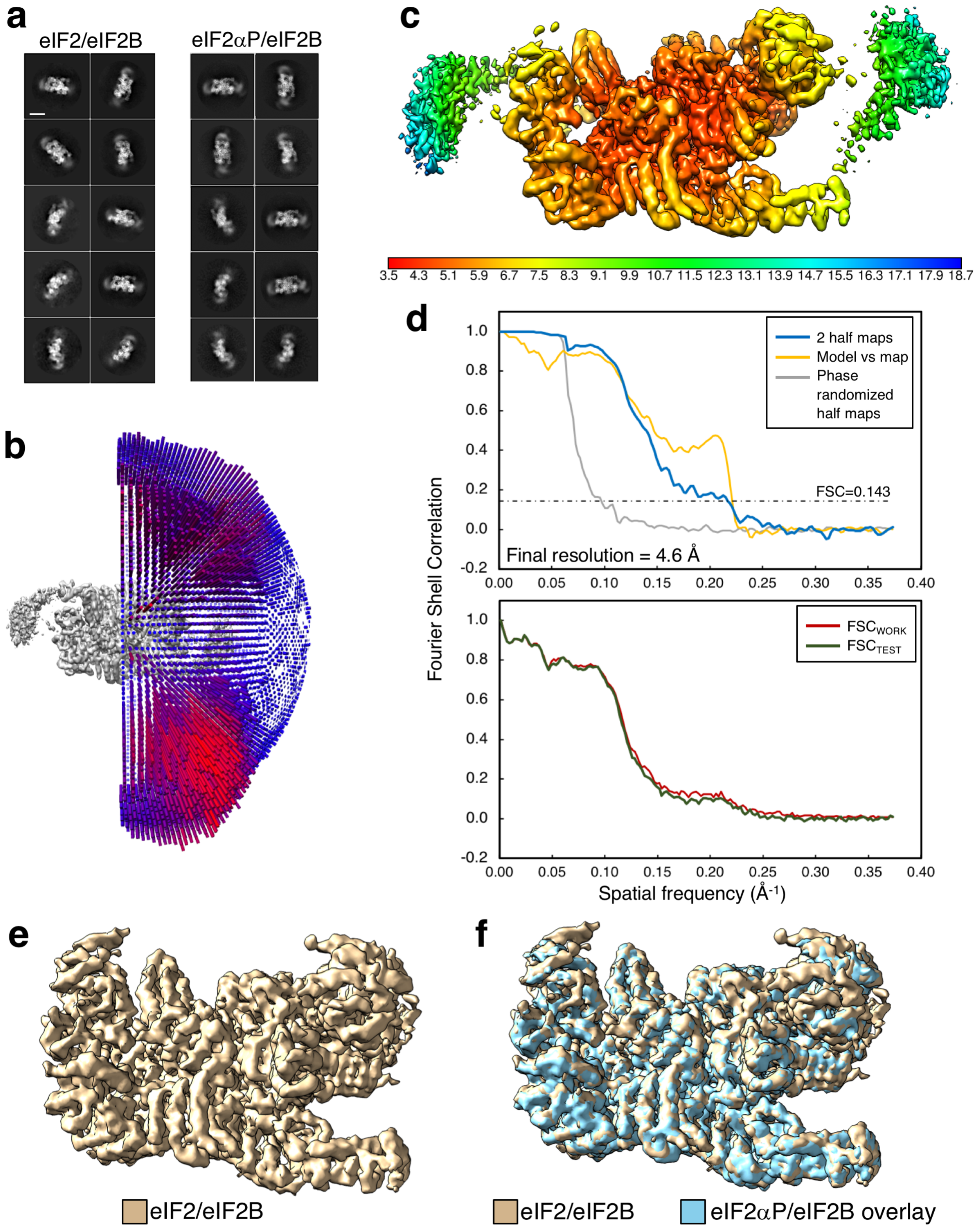

Supplementary Figure 6. Overview of eIF2/eIF2B structure

a, Representative 2D classes of the eIF2/eIF2B complex (Left) alongside similar classes selected from those shown in Supplementary Figure 2b. Scale bar 100 Å.
b, Particle angle plot. **c**, eIF2/eIF2B Locscale map showing local resolution as a color temperature scale (Å). **d**, FSC plots for eIF2/eIF2B, as Supplementary Figure 2f.
e, Masked core map eIF2/eIF2B density. **f**, Overlay of masked eIF2aP/eIF2B with radial Fourier amplitude profile re-scaled to match the eIF2/eIF2B map.

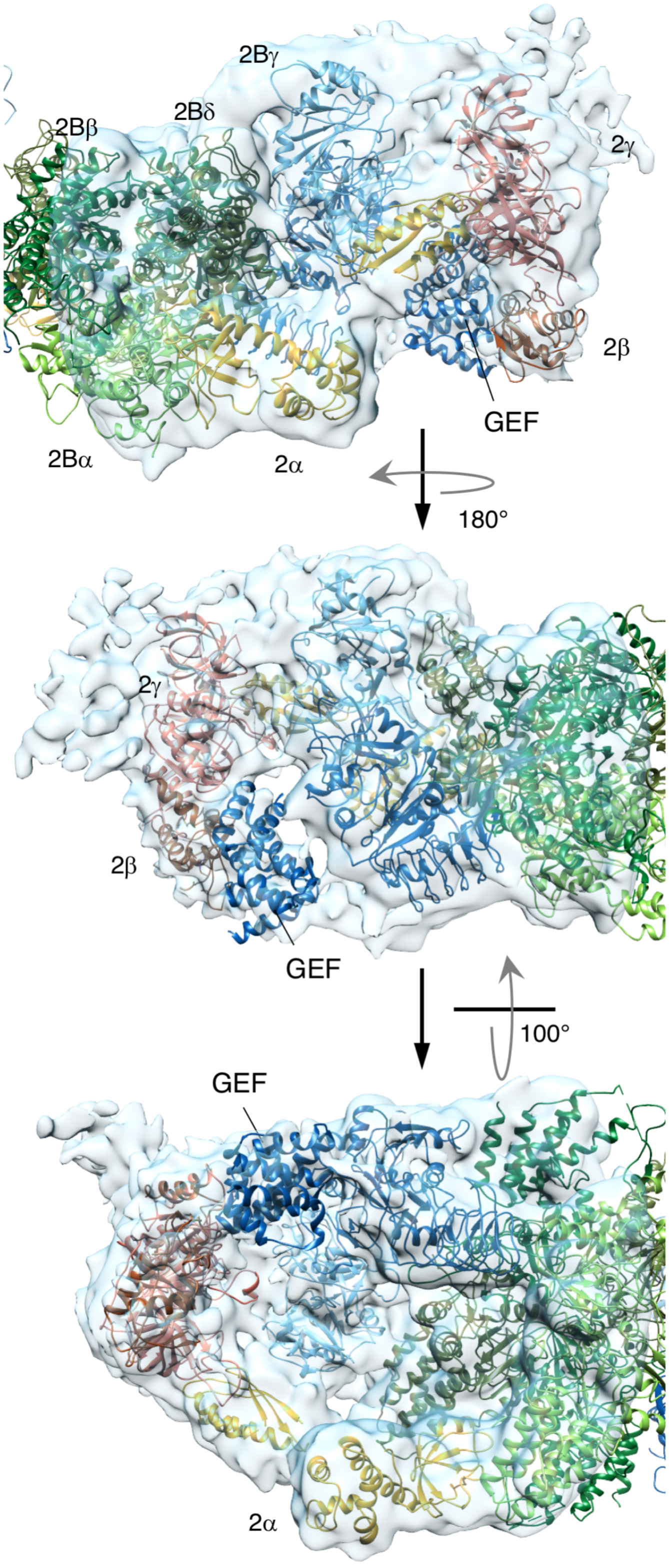

Supplementary Figure 7. Fitting eIF2β and eIF2Bε^{GEF}

Views of a 3D map from a particle class obtained when halves of the particles were independently classified using a localized reconstruction script described in the methods. Model coordinates from PDB files 3JAP (eIF2β) and 1PAQ (eIF2Bε^{GEF} domain) were fitted in the EM density.

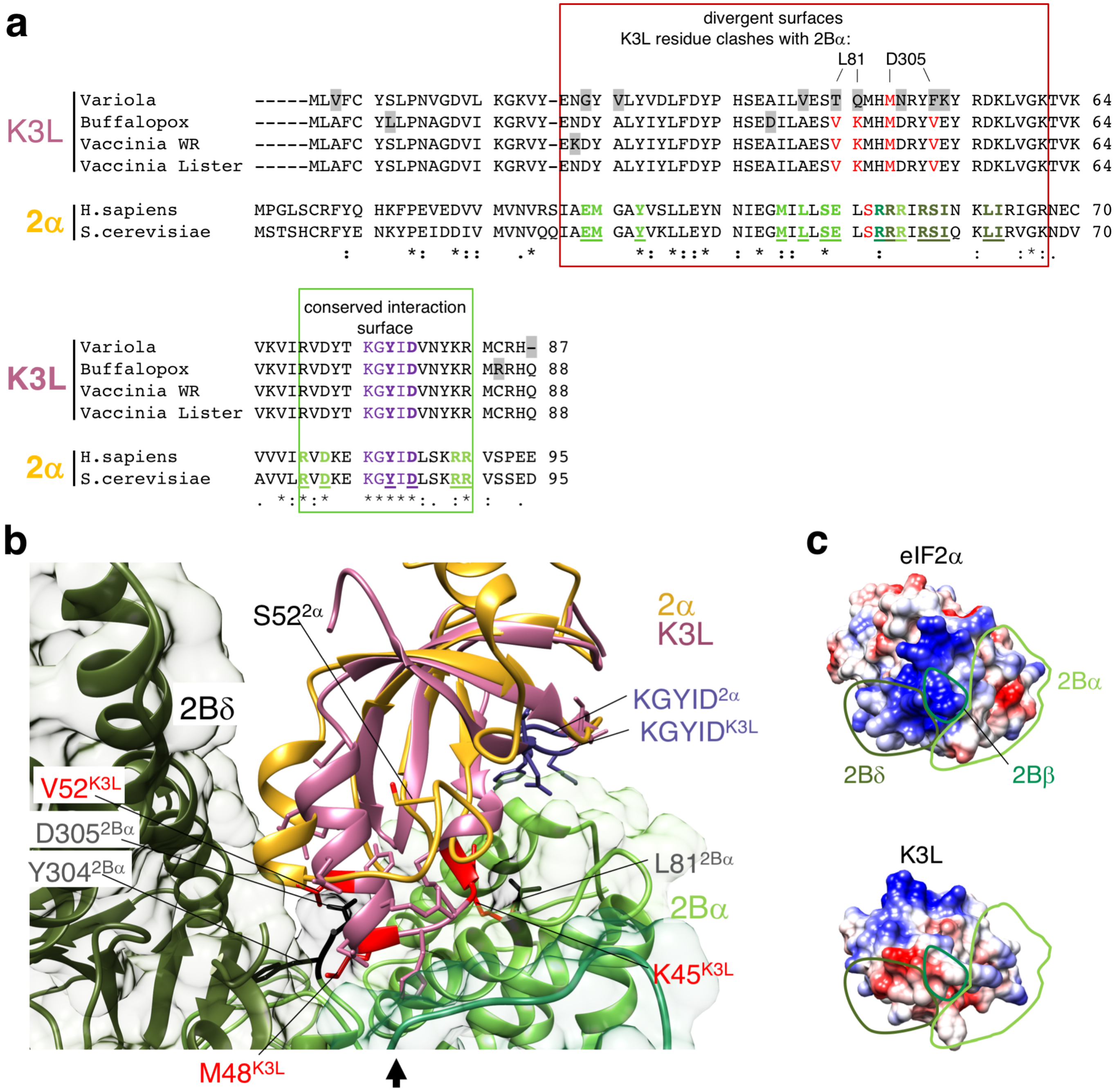

Supplementary Figure 8. Structural specificity of viral K3L for PKR over eIF2B

a, Alignment of K3L and eIF2α sequences. Sequence variation in K3L residues, highlighted grey. Residues colored red clash with eIF2Bα. eIF2α residues are colored by eIF2B interacting partner. K3L surface residues juxtaposed mainly to eIF2Bδ have distinct properties (red box) while 'KGYID' centered surface is conserved (green box).

b, Superposition of K3L (PDB:1LUZ; pink) on eIF2/eIF2B reveals clashes between K3L (red) and eIF2Bα residues (black) that should prevent K3L binding and inhibiting eIF2B.

c, eIF2α Coulombic surface potential at the eIF2B interface (viewed from direction of arrow in **b**), with eIF2B interaction zones indicated and shown below is the equivalent K3L surface.

Supplementary Tables

Supplementary Table 1. Data collection and reconstruction.

**Data collection**			
	**eIF2αP/eIF2B** (ultrathin carbon, 35° tilt)	**eIF2αP/eIF2B** (ice, no tilt)	**eIF2/eIF2B** (ultrathin carbon, 35° tilt)
Microscope	Titan Krios	Titan Krios	Titan Krios
Voltage (kV)	300	300	300
Detector	K2 Summit	K2 Summit	K2 Summit
Magnification (x)	37313	37313	37313
Pixel size (Å)	1.34	1.34	1.34
Defocus range (µm)	-1.5 to -3.5	-2 to -4	-1 to -3.0
Dose (e^-/Å^2)	40	30	34
Number of frames	48	40	40, 60, 100
Frame length (s)	0.25	0.15	0.35,0.23,0.14
Micrographs	2278	2255	2154

**Reconstruction**			
	**eIF2αP/eIF2B** (ultrathin carbon, 35° tilt)	**eIF2αP/eIF2B** (combined) EMDB-4404 PDB-613M	**eIF2/eIF2B** (ultrathin carbon, 35° tilt) EMDB-4428 PDB-617T
Initial particle images	196,242	50,000+42,622	114,390
Particles used for final refinement	42,622	64,541	23,274
Symmetry imposed	C2	C2	C2
Map resolution (Å)	4.3	3.9	4.6
Map resolution range (Å) (in masked core region)		3.5-6	4.1-8
Map resolution range (Å) (whole structure)		3.5-18	4.1-17
Fourier shell correlation criterion	0.143	0.143	0.143
Map sharpening B-factor (Å^2)	-138.1	-118.2	-151.4

Supplementary Table 2. Residues modelled and initial templates used for the eIF2αP/eIF2B complex (PDB-613M, EMDB-4404)****.**

Subunit	Residues total	Residues modelled	Initial template	Species
eIF2Bα	305	1-305	5B04A	*S. pombe*
eIF2Bβ	381	16-129<br>142-381	5B04C	*S. pombe*
eIF2Bγ*	578	2-10<br>67-88<br>107-309<br>382-415	5B04E	*S. pombe*
eIF2Bδ	651	247-534<br>598-651	5B04G	*S. pombe*
eIF2Bε	712	24-433	5B04I	*S. pombe*
eIF2α	304	3-174<br>182-210 (rigid fit)<br>218-265 (rigid fit)	3JAPj	*S. cerevisiae*
eIF2β	285	127-143 (rigid fit)	3JAPl	*S. cerevisiae*
eIF2γ	527	98-152 (rigid fit)<br>169-361 (rigid fit)<br>368-444 (rigid fit)<br>449-519 (rigid fit)	3JAPk	*S. cerevisiae*

*majority of this subunit is modelled into lower resolution map and relied almost exclusively on homology modelling and refinement in phenix.real_space_refine, therefore may not be accurate

** **eIF2/eIF2B** (PDB-617T, EMDB-4428) was refined from PDB-613M.

Supplementary Table 3. Modelling statistics.

	**eIF2αP/eIF2B**<br>PDB-6I3M<br>EMDB-4404	**eIF2/eIF2B**<br>PDB-6I7T<br>EMDB-4428
**Core Only**		
MolProbity score	1.96	2.00
Clashscore	6.82	7.19
Poor rotamers (%)	0.54	0.18
RMS bond lengths (Å)	0.0068	0.0052
RMS angles (degrees)	1.39	1.30
Ramachandran favoured (%)	88.72	87.64
Ramachandran allowed (%)	11.38	12.25
Ramachandran disallowed (%)	0.00	0.11
Model resolution (Å)	3.9	4.5
Model resolution range (Å)	3.5-6	4.1-8
Fourier shell correlation criterion	0.143	0.143
Total non-hydrogen atoms directly modelled	29,486	29,478
Total of residues directly modelled	3,702	3,702
**Final Model (incl rigid body docking)**		
Total non-hydrogen atoms modelled	37,012	37,004
Total residues modelled	4,682	4,682

Supplementary Table 4. RMSD similarity between eIF2α domains 1 and 2 in the eIF2αP/eIF2B structure and other eIF2α containing PDB entries

eIF2α chain compared*	*S. cer* eIF2αP/ eIF2B	*S. cer* eIF2/ eIF2B	*S. cer* 1Q46	*S. cer* 3JAP-j	*S. cer* 3JAQ-j	*S. cer* 3J81-j	human 6FEC-P	rabbit 5K0Y-P	residues aligned
Residues 3-173 (domains 1 and 2)									
eIF2αP/eIF2B	0	0.803	1.416	1.452	1.897	1.980	2.546	2.496	149
consensus	1.049	1.188	1.248	1.248	1.304	1.448	1.853	1.827	149
1Q46	1.416	1.612	0	1.629	1.765	2.181	2.542	2.656	149
Residues 3-88 (domain 1 only)									
eIF2αP/eIF2B	0	0.509	1.393	1.542	1.627	1.624	2.166	2.063	75
consensus	0.983	0.950	1.075	1.170	1.123	1.036	1.477	1.472	75
1Q46	1.393	1.349	0	1.759	1.670	1.676	2.001	1.967	75

* RMSD differences (Å) when compared with these reference structures

Supplementary Table 5. Similarity between eIF2B in the eIF2αP/eIF2B structure and other eIF2B decamer structures

Per subunit RMSD (Å)					
eIF2B chain	*S. cer* eIF2αP/eIF2B	*S. cer* eIF2/eIF2B	*S. pombe* 5B04	human 6CAJ	human 6EZO
alpha	0	0.573	1.569	1.396	1.335
beta	0	0.601	1.323	1.570	1.637
gamma	0	1.002	2.573	2.542	2.656
delta	0	0.638	1.470	1.588	1.693
epsilon	0	0.630	1.490	1.490	1.497

Sequence Identity					
chain	*S. cer* eIF2αP/eIF2B	*S. cer* eIF2/eIF2B	*S. pombe* 5B04	human 6CAJ	human 6EZO
alpha	1	1	0.484	0.445	0.453
beta	1	1	0.445	0.365	0.368
gamma	1	1	0.296	0.180	0.214
delta	1	1	0.447	0.425	0.399
epsilon	1	1	0.507	0.345	0.326

No. aa in structure						
chain	*S. cer* eIF2αP/eIF2B	*S. cer* eIF2/eIF2B	*S. pombe* 5B04	human 6CAJ	human 6EZO	Cα atoms aligned
alpha	305	305	317	265	280	254
beta	354	354	349	318	307	299
gamma	268	268	384	244	347	206
delta	342	342	349	357	355	318
epsilon	410	410	428	421	396	377

Supplementary Discussion

Comparison of our eIF2/eIF2B structures with cross-linking data and other recent structures.

Kashiwagi et al., 2016 published an extensive series of protein-protein interaction cross-links between specific eIF2B subunit residues and eIF2 and *vice versa* using *S. pombe* proteins. These data showed cross-links between eIF2α and eIF2Bα, 2Bβ and 2Bδ and between eIF2γ and eIF2Bγ and eIF2Bε[1]. By reciprocal labelling of specific residues in one binding partner, they built a picture of the interaction interfaces between eIF2 and eIF2B. In large agreement with our structures, their data showed that the cross-links were largely unchanged by the phosphorylation of eIF2α at serine 52[1].

All eIF2Bαβδ cross-links examined (bar two eIF2Bβ positions) were found unaffected by loss of phosphate from eIF2αP. In our structures the eIF2Bβ density is weaker and contributes less to the interaction interface (Supplementary Figure 4c). Reciprocal cross-links identified between labelled eIF2α and eIF2Bα are also consistent with our structures. However, the reciprocal eIF2α-eIF2Bβ cross-links are not[1]. For example *S. pombe* eIF2α-R88 cross-links to both eIF2Bα and eIF2Bβ. In our structures the equivalent residue (eIF2α-R89) is close to eIF2Bα, but not eIF2Bβ (Supplementary Figure 4c; see also paragraph below). The cross-links between eIF2Bγ or eIF2Bε and eIF2γ are also largely unchanged by eIF2αP, where both forms were analysed and are consistent with our structure[1]. Our resolution of eIF2γ is poor, because eIF2γ can adopt multiple positions (Figure 2). This limits detailed comparison between specific cross-links and our structure. Our structures place eIF2γ closer to eIF2Bγ than eIF2Bε. Interestingly, the *S. pombe* cross-links between eIF2BγS258 and eIF2γ were stronger that those between the eIF2Bε positions analysed and eIF2γ[1]. These comparisons suggest that our structures are consistent with the majority of cross-links previously described.

Concurrent independent studies have also determined the structures of eIF2B bound to eIF2 and for which pre-prints are deposited in BioRxiv[2-4]. Each study performs a range of complementary experiments. All are broadly agreed in how eIF2B interacts with phosphorylated eIF2 via the eIF2α-eIF2Bα/δ/β interface. One study additionally shows binding of *S. pombe* eIF2B to unphosphorylated eIF2α from *S. cerevisiae* in an identical

position to our study[4]. In contrast, two studies show human eIF2 interacting with human eIF2B at a different site where eIF2α is bound between eIF2Bβ and eIF2Bδ and not via eIF2Bα[3,4]. Although we do not have evidence for this conformation in our cryoEM images, the binding mode described does appear consistent with the prior eIF2Bβ cross-link to *S. pombe* eIF2α-R88 discussed above, that is not in common with our structure. The alternative binding site would not be expected to allow the cross-links seen to eIF2Bα. Taken together the data suggest that two alternative binding modes between eIF2 and eIF2B may be possible. The implications of this for GEF activity and regulation in the ISR are not yet clear and will merit further study.

Supplementary References

1. Kashiwagi, K. et al. Crystal structure of eukaryotic translation initiation factor 2B. *Nature* **531**, 122-125 (2016).
2. Gordiyenko, Y., Llacer, J.L. & Ramakrishnan, V. Structural basis for the inhibition of translation through eIF2α phosphorylation. P reprint at http://dx.doi.org/10.1101/503979 (2018).
3. Anand, A.A. et al. Structural basis of eIF2B-catalyzed GDP exchange and phosphoregulation by the integrated stress response. Preprint at http://dx.doi.org/10.1101/504654 (2018).
4. Kashiwagi, K. et al. Structural Basis for eIF2B Inhibition in Integrated Stress Response. Preprint at http://dx.doi.org/10.1101/503540 (2018).
