# Supplementary material for: The structural basis of translational control by eIF2 phosphorylation
Source: Nat Commun. 2019 May 13;10:2136. doi: 10.1038/s41467-019-10167-3 (PMC6513899; doi:10.1038/s41467-019-10167-3)
Supplement: Supplementary file 3 — Reporting Summary [file 41467_2019_10167_MOESM3_ESM.pdf]

nature research

Corresponding author(s): Graham Pavitt, Alan Roseman

Last updated by author(s): Dec 29, 2018

Reporting Summary

Nature Research wishes to improve the reproducibility of the work that we publish. This form provides structure for consistency and transparency in reporting. For further information on Nature Research policies, see Authors & Referees and the Editorial Policy Checklist.

Statistics

For all statistical analyses, confirm that the following items are present in the figure legend, table legend, main text, or Methods section.

n/a	Confirmed	
☐	☒	The exact sample size (*n*) for each experimental group/condition, given as a discrete number and unit of measurement
☐	☒	A statement on whether measurements were taken from distinct samples or whether the same sample was measured repeatedly
☒	☐	The statistical test(s) used AND whether they are one- or two-sided<br>*Only common tests should be described solely by name; describe more complex techniques in the Methods section.*
☒	☐	A description of all covariates tested
☒	☐	A description of any assumptions or corrections, such as tests of normality and adjustment for multiple comparisons
☒	☐	A full description of the statistical parameters including central tendency (e.g. means) or other basic estimates (e.g. regression coefficient) AND variation (e.g. standard deviation) or associated estimates of uncertainty (e.g. confidence intervals)
☒	☐	For null hypothesis testing, the test statistic (e.g. *F*, *t*, *r*) with confidence intervals, effect sizes, degrees of freedom and *P* value noted<br>*Give P values as exact values whenever suitable.*
☒	☐	For Bayesian analysis, information on the choice of priors and Markov chain Monte Carlo settings
☒	☐	For hierarchical and complex designs, identification of the appropriate level for tests and full reporting of outcomes
☒	☐	Estimates of effect sizes (e.g. Cohen's *d*, Pearson's *r*), indicating how they were calculated

Our web collection on statistics for biologists contains articles on many of the points above.

Software and code

Policy information about availability of computer code

Data collection	Automated cryoEM data collection was performed using EPU software from Thermo Fisher Scientific, on a Titan Krios 300kV TEM equipped with a Gatan K2 Summit Detector, imaging at 37313x magnification.
Data analysis	CryoEM data were analyzed using the software dosefgpu driftcorr, MotionCor2, CTFFIND4, Gctf, cryoSPARC, RELION 1.4, RELION 2.1, ResMap, 3Dradamp and LOCSCALE. Model building and refinement were performed using MODELLER, UCSF Chimera, COOT and PHENIX Visualization was performed with COOT and UCSF Chimera. Atomic coordinate alignment and analysis were performed using GESAMT. Western Blots were analyzed by Li-Cor Image Studio software.

For manuscripts utilizing custom algorithms or software that are central to the research but not yet described in published literature, software must be made available to editors/reviewers. We strongly encourage code deposition in a community repository (e.g. GitHub). See the Nature Research guidelines for submitting code & software for further information.

Data

Policy information about availability of data

All manuscripts must include a data availability statement. This statement should provide the following information, where applicable:

- Accession codes, unique identifiers, or web links for publicly available datasets
- A list of figures that have associated raw data
- A description of any restrictions on data availability

The cryoEM density maps and atomic coordinates have been deposited in the Electron Microscopy Data Bank and the Protein Data Bank, under accessions EMD-4404 and 6I3M, respectively for the eIF2alphaP/eIF2B complex and EMD-4428 and 6I7T for the eIF2/eIF2B All other data are available upon request to the corresponding authors.

Field-specific reporting

Please select the one below that is the best fit for your research. If you are not sure, read the appropriate sections before making your selection.

☒ Life sciences ☐ Behavioural & social sciences ☐ Ecological, evolutionary & environmental sciences

For a reference copy of the document with all sections, see nature.com/documents/nr-reporting-summary-flat.pdf

Life sciences study design

All studies must disclose on these points even when the disclosure is negative.

Sample size	The amount of cryoEM data collected was limited by the access time to the microscopes at the CryoEM facilities at the UK national Electron Bio-Imaging Centre (eBIC).
Data exclusions	No data were excluded.
Replication	For PKR/eIF2B competion assay. 4 replicates were done each with a single PKR concentration and a range of eIF2B concentrations. The same trend was shown in each replicate.
Randomization	Samples were not allocated into groups. Randomization is not relevant to this study.
Blinding	No blinding was performed as it is not relevant to this study.

Reporting for specific materials, systems and methods

We require information from authors about some types of materials, experimental systems and methods used in many studies. Here, indicate whether each material, system or method listed is relevant to your study. If you are not sure if a list item applies to your research, read the appropriate section before selecting a response.

Materials & experimental systems

n/a	Involved in the study	
☐	☒	Antibodies
☒	☐	Eukaryotic cell lines
☒	☐	Palaeontology
☒	☐	Animals and other organisms
☒	☐	Human research participants
☒	☐	Clinical data

Methods

n/a	Involved in the study	
☒	☐	ChIP-seq
☒	☐	Flow cytometry
☒	☐	MRI-based neuroimaging

Antibodies

Antibodies used	Anti-yeast eIF2alpha (SUI2) raised in rabbit and for one blot raised in chickens to His-tagged SUI2 expressed and purified from E. coli and affinity purified. Phospho-specific eIF2alpha antibody was from CST, Antibody #9721-rabbit antibody.
Validation	Chicken antibody was validated in-house using yeast strains expressing eIF2 alpha and with purified eIF2 samples.
