# Supplementary material for: The structural basis of translational control by eIF2 phosphorylation
Source: Nat Commun. 2019 May 13;10:2136. doi: 10.1038/s41467-019-10167-3 (PMC6513899; doi:10.1038/s41467-019-10167-3)
Supplement: Supplementary file 4 — Description of Additional Supplementary Information [file 41467_2019_10167_MOESM4_ESM.pdf]

Description of Additional Supplementary Files

File Name: Supplementary Movie 1
Description: **Simulated movement in eIF2 from distinct domain orientations.** Related to Figure 2, simulated movement from half-map re-classifications of eIF2αP/eIF2B complexes.

File Name: Supplementary Movie 2
Description: **Model for coupled GDP exchange and tRNAi binding to form TC.** Movie related to model scheme shown in Figure 6. Shows eIF2 binding and eIF2B GEF activity. It combines the eIF2α domain 3 movements shown in Figure 2 and Supplementary Movie 1 with the location of the GEF domain shown in Supplementary Figure 7. Initiator tRNA can bind to form TC while eIF2 is bound to eIF2B in an extended conformation, coupling nucleotide exchange and TC formation.
